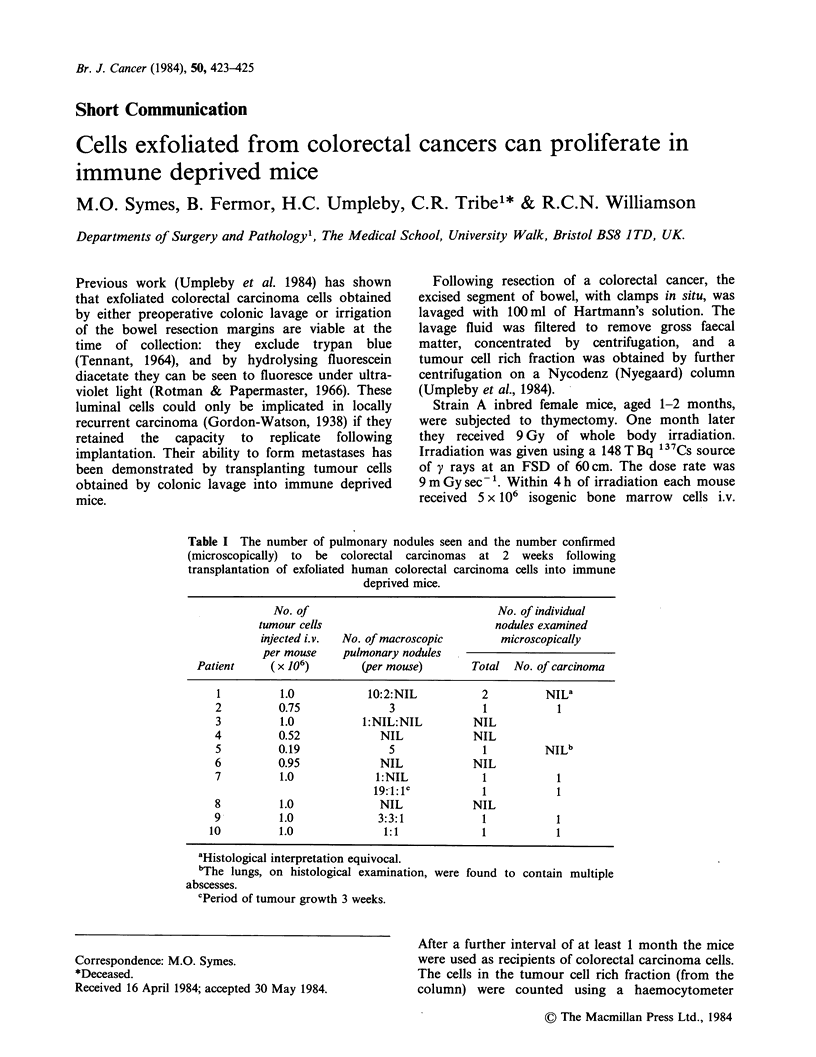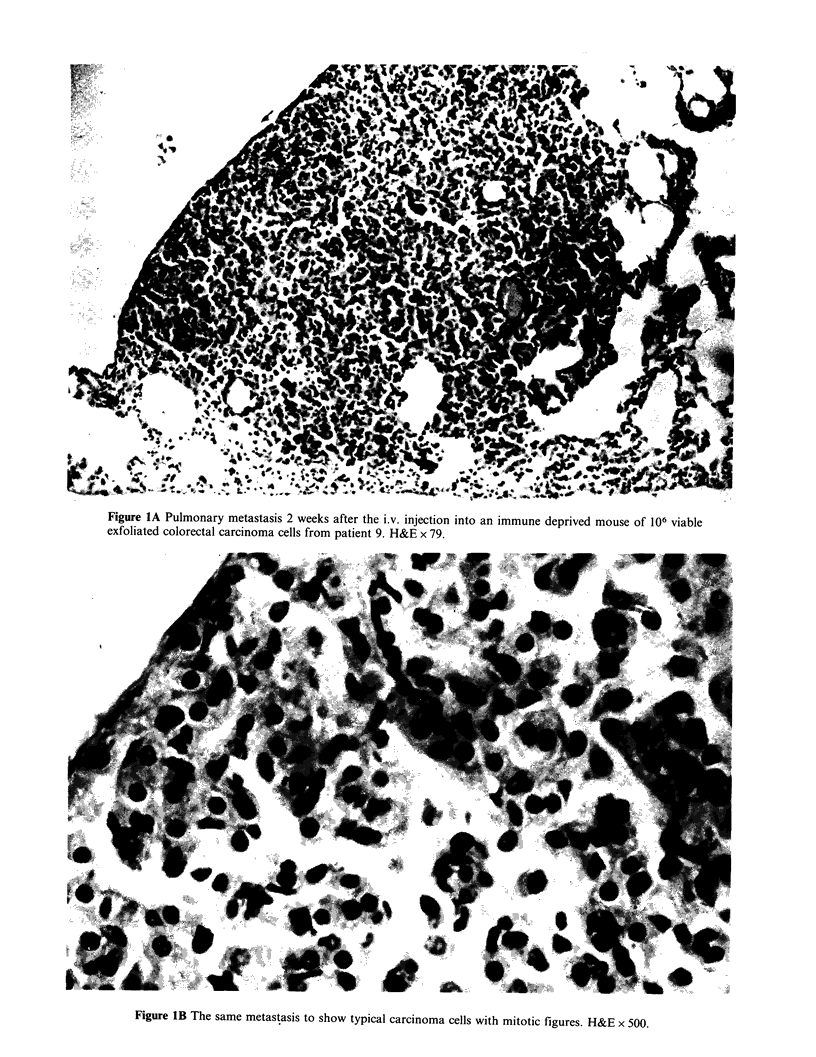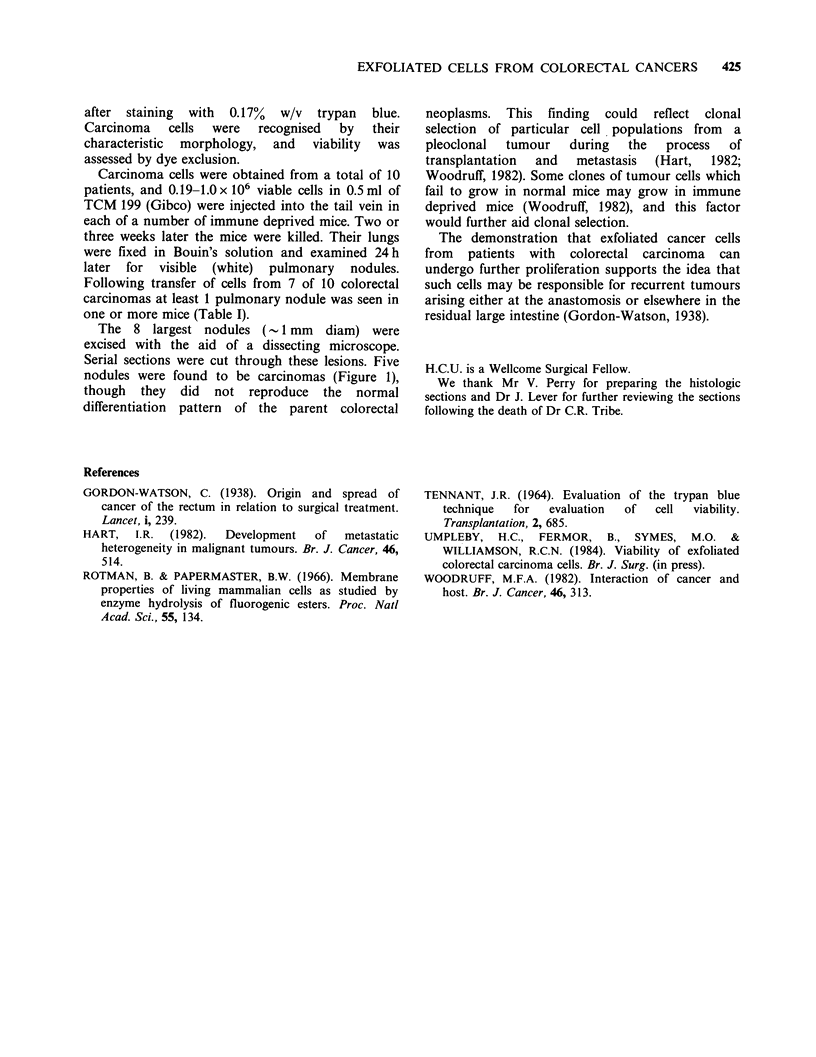# Cells exfoliated from colorectal cancers can proliferate in immune deprived mice.

**DOI:** 10.1038/bjc.1984.193

**Published:** 1984-09

**Authors:** M. O. Symes, B. Fermor, H. C. Umpleby, C. R. Tribe, R. C. Williamson

## Abstract

**Images:**


*Br. J. Cancer* (1984), **50**, 423–425

### Short Communication

### Cells exfoliated from colorectal cancers can proliferate in immune deprived mice

M.O. Symes, B. Fermor, H.C. Umpleby, C.R. Tribe[1]* & R.C.N. Williamson

*Departments of Surgery and Pathology[1], The Medical School, University Walk, Bristol BS8 1TD, UK.*

Previous work (Umpleby *et al.* 1984) has shown that exfoliated colorectal carcinoma cells obtained by either preoperative colonic lavage or irrigation of the bowel resection margins are viable at the time of collection: they exclude trypan blue (Tennant, 1964), and by hydrolysing fluorescein diacetate they can be seen to fluoresce under ultraviolet light (Rotman & Papermaster, 1966). These luminal cells could only be implicated in locally recurrent carcinoma (Gordon-Watson, 1938) if they retained the capacity to replicate following implantation. Their ability to form metastases has been demonstrated by transplanting tumour cells obtained by colonic lavage into immune deprived mice.

Following resection of a colorectal cancer, the excised segment of bowel, with clamps *in situ*, was lavaged with 100 ml of Hartmann's solution. The lavage fluid was filtered to remove gross faecal matter, concentrated by centrifugation, and a tumour cell rich fraction was obtained by further centrifugation on a Nycodenz (Nyegaard) column (Umpleby *et al.*, 1984).

Strain A inbred female mice, aged 1–2 months, were subjected to thymectomy. One month later they received 9 Gy of whole body irradiation. Irradiation was given using a 148 T Bq $^{137}$Cs source of $\gamma$ rays at an FSD of 60 cm. The dose rate was 9 m Gy sec$^{-1}$. Within 4 h of irradiation each mouse received $5 \times 10^6$ isogenic bone marrow cells i.v. After a further interval of at least 1 month the mice were used as recipients of colorectal carcinoma cells. The cells in the tumour cell rich fraction (from the column) were counted using a haemocytometer

**Table I** The number of pulmonary nodules seen and the number confirmed (microscopically) to be colorectal carcinomas at 2 weeks following transplantation of exfoliated human colorectal carcinoma cells into immune deprived mice.

| Patient | *No. of tumour cells injected i.v. per mouse* ($\times 10^6$) | *No. of macroscopic pulmonary nodules (per mouse)* | *No. of individual nodules examined microscopically* | |
|---|---|---|---|---|
| | | | *Total* | *No. of carcinoma* |
| 1 | 1.0 | 10:2:NIL | 2 | NIL[a] |
| 2 | 0.75 | 3 | 1 | 1 |
| 3 | 1.0 | 1:NIL:NIL | NIL | |
| 4 | 0.52 | NIL | NIL | |
| 5 | 0.19 | 5 | 1 | NIL[b] |
| 6 | 0.95 | NIL | NIL | |
| 7 | 1.0 | 1:NIL | 1 | 1 |
| | | 19:1:1[c] | 1 | 1 |
| 8 | 1.0 | NIL | NIL | |
| 9 | 1.0 | 3:3:1 | 1 | 1 |
| 10 | 1.0 | 1:1 | 1 | 1 |

[a]Histological interpretation equivocal.
[b]The lungs, on histological examination, were found to contain multiple abscesses.
[c]Period of tumour growth 3 weeks.

Correspondence: M.O. Symes.
*Deceased.
Received 16 April 1984; accepted 30 May 1984.

© The Macmillan Press Ltd., 1984

**Figure 1A** Pulmonary metastasis 2 weeks after the i.v. injection into an immune deprived mouse of $10^6$ viable exfoliated colorectal carcinoma cells from patient 9. H&E × 79.

**Figure 1B** The same metastasis to show typical carcinoma cells with mitotic figures. H&E × 500.

after staining with 0.17% w/v trypan blue. Carcinoma cells were recognised by their characteristic morphology, and viability was assessed by dye exclusion.

Carcinoma cells were obtained from a total of 10 patients, and 0.19–1.0 × $10^6$ viable cells in 0.5 ml of TCM 199 (Gibco) were injected into the tail vein in each of a number of immune deprived mice. Two or three weeks later the mice were killed. Their lungs were fixed in Bouin's solution and examined 24 h later for visible (white) pulmonary nodules. Following transfer of cells from 7 of 10 colorectal carcinomas at least 1 pulmonary nodule was seen in one or more mice (Table I).

The 8 largest nodules (~1 mm diam) were excised with the aid of a dissecting microscope. Serial sections were cut through these lesions. Five nodules were found to be carcinomas (Figure 1), though they did not reproduce the normal differentiation pattern of the parent colorectal neoplasms. This finding could reflect clonal selection of particular cell populations from a pleoclonal tumour during the process of transplantation and metastasis (Hart, 1982; Woodruff, 1982). Some clones of tumour cells which fail to grow in normal mice may grow in immune deprived mice (Woodruff, 1982), and this factor would further aid clonal selection.

The demonstration that exfoliated cancer cells from patients with colorectal carcinoma can undergo further proliferation supports the idea that such cells may be responsible for recurrent tumours arising either at the anastomosis or elsewhere in the residual large intestine (Gordon-Watson, 1938).

H.C.U. is a Wellcome Surgical Fellow.

We thank Mr V. Perry for preparing the histologic sections and Dr J. Lever for further reviewing the sections following the death of Dr C.R. Tribe.

### References

GORDON-WATSON, C. (1938). Origin and spread of cancer of the rectum in relation to surgical treatment. *Lancet*, **i**, 239.

HART, I.R. (1982). Development of metastatic heterogeneity in malignant tumours. *Br. J. Cancer*, **46**, 514.

ROTMAN, B. & PAPERMASTER, B.W. (1966). Membrane properties of living mammalian cells as studied by enzyme hydrolysis of fluorogenic esters. *Proc. Natl Acad. Sci.*, **55**, 134.

TENNANT, J.R. (1964). Evaluation of the trypan blue technique for evaluation of cell viability. *Transplantation*, **2**, 685.

UMPLEBY, H.C., FERMOR, B., SYMES, M.O. & WILLIAMSON, R.C.N. (1984). Viability of exfoliated colorectal carcinoma cells. *Br. J. Surg*. (in press).

WOODRUFF, M.F.A. (1982). Interaction of cancer and host. *Br. J. Cancer*, **46**, 313.